# MRI anatomy of the rectum: key concepts important for rectal cancer staging and treatment planning

**DOI:** 10.1186/s13244-022-01348-8

**Published:** 2023-01-18

**Authors:** Nino Bogveradze, Petur Snaebjornsson, Brechtje A. Grotenhuis, Baukelien van Triest, Max J. Lahaye, Monique Maas, Geerard L. Beets, Regina G. H. Beets-Tan, Doenja M. J. Lambregts

**Affiliations:** 1 Department of Radiology, The Netherlands Cancer Institute, P.O. Box 90203, 1006 BE Amsterdam, The Netherlands; 2 GROW School for Oncology and Developmental Biology, University of Maastricht, Maastricht, The Netherlands; 3 Department of Radiology, American Hospital Tbilisi, Tbilisi, Georgia; 4 Department of Pathology, Netherlands Cancer Institute, Amsterdam, The Netherlands; 5 Department of Surgery, Netherlands Cancer Institute, Amsterdam, The Netherlands; 6 Department of Radiation Oncology, Netherlands Cancer Institute, Amsterdam, The Netherlands; 7 Institute of Regional Health Research, University of Southern Denmark, Odense, Denmark

**Keywords:** Magnetic resonance imaging, Rectal cancer, Anatomy, Staging, Treatment planning

## Abstract

A good understanding of the MRI anatomy of the rectum and its surroundings is pivotal to ensure high-quality diagnostic evaluation and reporting of rectal cancer. With this pictorial review, we aim to provide an image-based overview of key anatomical concepts essential for treatment planning, response evaluation and post-operative assessment. These concepts include the cross-sectional anatomy of the rectal wall in relation to T-staging; differences in staging and treatment between anal and rectal cancer; landmarks used to define the upper and lower boundaries of the rectum; the anatomy of the pelvic floor and anal canal, the mesorectal fascia, peritoneum and peritoneal reflection; and guides to help discern different pelvic lymph node stations on MRI to properly stage regional and non-regional rectal lymph node metastases. Finally, this review will highlight key aspects of post-treatment anatomy, including the assessment of radiation-induced changes and the evaluation of the post-operative pelvis after different surgical resection and reconstruction techniques.

## Key points

- MRI plays a key role in the staging and treatment stratification of rectal cancer.
- Understanding rectal and pelvic MRI anatomy is pivotal for high-quality reporting.
- This review addresses key anatomy concepts for staging, treatment planning, and follow-up.

## Background

Magnetic resonance imaging (MRI) plays a key role in the staging and treatment stratification of patients with rectal cancer. High-resolution MRI can accurately assess tumour infiltration in and beyond different layers of the bowel wall, as well as invasion into important anatomical structures such as the mesorectal fascia (MRF), peritoneum, and surrounding pelvic organs [1–6]. By doing so, MRI provides crucial information to determine the risk profile of each individual patient to help decide who will benefit from neoadjuvant (chemo)radiotherapy (CRT) [6, 7]. The MRI findings can guide the surgical approach and help the radiation oncologist to accurately define his radiation target volumes. MRI has also been widely adopted as a valuable tool to assess response after neoadjuvant treatment. The findings of restaging MRI can help the surgeon to fine tune his surgical approach and aid in the selection of patients with a (near) complete response who may be candidates for organ-preserving treatment alternatives such as watch-and-wait (W&W) [2, 8–10]. MRI is also valuable to help determine the local extent of disease in case of a suspected pelvic recurrence [11–13]. In all these scenarios, a good understanding of the MRI anatomy of the rectum and its surroundings is pivotal to ensure high-quality diagnostic evaluation and reporting. This pictorial review will discuss key anatomical concepts essential for staging, treatment planning, and post-treatment assessment on MRI.

## The rectal wall and T-staging

**Fig. 1:**
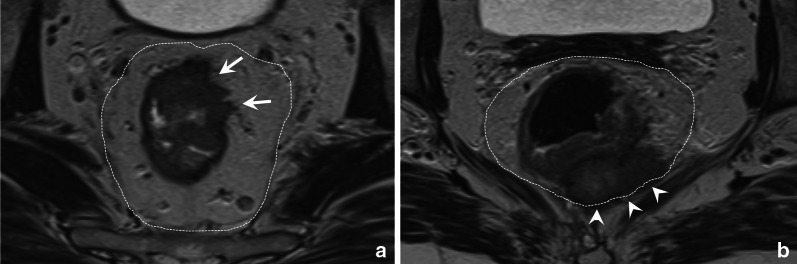
Axial T2-weighted images of a low risk T3ab tumour without MRF involvement (**a**) and a high-risk T3cd tumour with MRF involvement (**b**). The left patient is a 52 year old male patient with a tumour that extends beyond the muscularis propria from approximately 12 till 2 o’clock (white arrows) with an extramural invasion depth of < 5 mm. There is a sufficient margin (> 1 mm) between the tumour and MRF. The right patient is a 55-year-old female patient with extensive extramural invasion from 4 to 6 o’clock with broad-based involvement of the MRF (white arrowheads)

Anatomically, the rectal wall comprises three main layers: an inner mucosal layer, the underlying submucosa, and an outer muscular layer, the muscularis propria. The depth of invasion into and beyond these layers determines the tumour stage (T-stage) in rectal cancer, as outlined in Table 1 [4, 15, 26]. T-stage is one of the prognostic risk factor used in clinical guidelines to determine the most appropriate treatment strategy. Tumours that remain limited to the submucosa (T1) or that extend into but not beyond the muscularis propria (T2) are typically considered clinically as early-stage tumours that may be managed with surgery only (total mesorectal excision) or even local endoscopic excision [4, 14, 15], provided that there are no other adverse features such as lymph node metastases. Tumours that grow beyond the muscularis propria into the mesorectal fat, i.e. tumours with extramural invasion, are classified as T3. These can range from low-risk tumours with limited extramural invasion (T3a and T3b) to more high-risk tumours with more extensive extramural invasion (T3c and T3d), or T3 tumours that invade the MRF (see Fig. 1 and also section on *mesorectum and mesorectal fascia* below) [4, 15]. These high-risk tumours typically require neoadjuvant (chemo)radiotherapy [4].

**Table 1:** Tumour (T) staging in rectal cancer

T1	Tumour invades submucosa
T2	Tumour invades muscularis propria
T3	Tumour invades through muscularis propria into perirectal fat
	T3a: < 1 mm T3b 1–5 mm T3c > 5–15 mm T3d > 15 mm
T4a	Tumour invades peritoneum or peritoneal reflection
T4b	Tumour invades adjacent organs or structures^a^
	Bone, striated muscle (incl. external anal sphincter, pelvic floor, piriformis), ureters, urethra, nerves, vessels outside mesorectal compartment, any loop of small/large bowel other than loop from which the tumour originates, any fat in anatomical compartment outside the mesorectum (obturator, para-iliac, ischiorectal space)

^a^Definitions for structures to be included in the definition of T4b disease were derived from a recent publication by Lambregts et al. on controversies in TNM staging [26]

Further definitions are derived from the 8th edition of the AJCC/UICC tumour node metastases (TNM) staging manual and the 2017 ESMO guidelines on the clinical management of rectal cancer [4, 15]

**Fig. 2:**
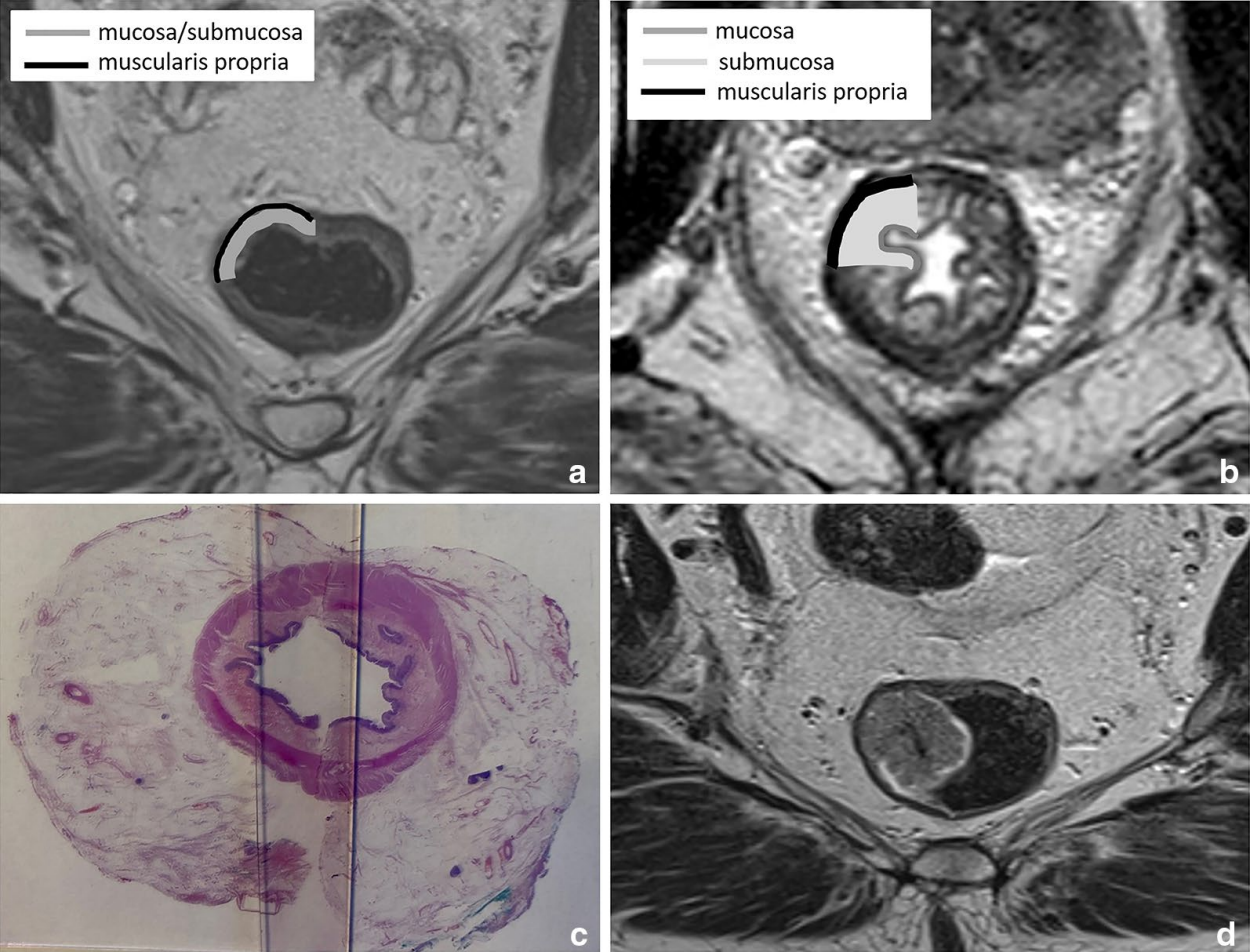
Examples showing the normal two-layered (**a**) versus oedematous three-layered (**b**) appearance of the rectal wall on axial T2-weighted MRI and the corresponding cross-sectional wall anatomy at histopathology (**c**). Figure **d** shows an example of a 63-year-old male rectal cancer patient with a polypoid tumour staged as cT1-2 considering that the submucosa is not separately visible, making it impossible to determine whether this tumour invades the submucosa (T1) or infiltrates the muscularis propria (T2)

It is important to realise that on a routine T2-weighted MRI, the rectal wall typically has a two-layered instead of three-layered appearance with a total thickness of only 2–3 mm [16]. The mucosa and submucosa are in most cases indistinguishable and seen as a single intermediate signal layer surrounded by a second T2-hypointense layer that represents the muscularis propria (Fig. 2A). The mucosa and submucosa can be recognised as separate layers on MRI in the presence of submucosal oedema (for example, as a result of radiation therapy). In these cases, the submucosa is visualised as a high signal middle layer between the mucosa and muscularis propria (Fig. 2B). The limited visibility of the separate layers of the bowel wall is one of the main reasons why MRI is generally unable to discern T1 from T2 tumours and why these are often reported together on MRI as stage cT1-2 (Fig. 2D), as is also the case in the structured reporting and staging template published by the European Society of Gastrointestinal and Abdominal Radiology (ESGAR) [2].

## Upper and lower boundaries of the rectum

### Upper boundary: the sigmoid take-off

**Fig. 3:**
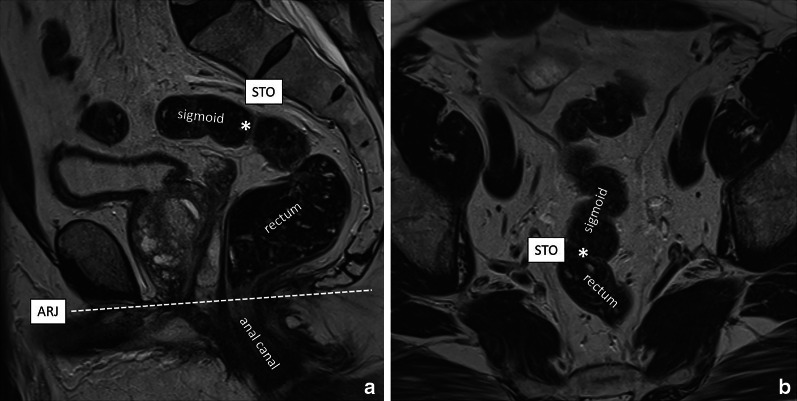
Sagittal and axial T2-weighted images of a normal rectum of a male individual (without rectal cancer) demonstrating the sigmoid take-off (STO, indicated by the *) as the point from which the sigmoid sweeps horizontally on a sagittal view (**a**) and ventrally on an axial view (**b**), away from the sacrum. The white dashed line on the sagittal view indicates the anorectal junction (ARJ) that is typically situated at the level of an imaginary line between the lower margin of the sacral and pubic bone

Various definitions and landmarks have been used throughout the years to determine the upper boundary of the rectum, including the sacral promontory, third sacral vertebra, anterior peritoneal reflection, and distance measurements from the anorectal junction or anal verge [4, 17, 18]. The main clinical significance of defining the upper limit of the rectum during tumour staging is to differentiate rectal tumours from tumours arising in the sigmoid colon. Patients with sigmoid cancers are primarily managed with upfront surgery, while patients with rectal cancers usually undergo differentiated treatments varying from surgery only in low-risk tumours to short or long course neoadjuvant (chemo)radiotherapy in intermediate and high-risk tumours [4, 14, 15]. Recently, an international multidisciplinary expert consensus panel agreed on the “sigmoid take-off” (STO) as the preferred landmark to define the boundary between the rectum and sigmoid colon on imaging [19]. The STO marks the junction between the mesorectum and sigmoid mesocolon and can be recognised on sagittal views as the point from which the sigmoid sweeps horizontally (away from the sacrum) and on axial views as the point from which the sigmoid projects ventrally (Fig. 3).

Recognising the STO on imaging requires some training. It may be challenging due to variations in the anatomical course of the rectosigmoid related to the degree of luminal distension (by tumour or gas), mass effect from adjacent organs, pelvic floor insufficiency, or surgical history. The inconsistent angulation of axial imaging planes on MRI may also be a challenging factor [20, 21]. Nevertheless, the STO is generally considered an intuitive landmark. In 2019, the Dutch guidelines on colorectal cancer were one of the first to adopt the STO as a formal landmark to discern rectal from sigmoid cancer, defining rectal cancer as any tumour with a lower boundary starting below the level of the STO and sigmoid cancer as any tumour situated entirely above the level of the STO [14]. Reports from the Netherlands have shown that this new definition can impact treatment planning (e.g. the choice of surgery or neoadjuvant treatment) compared to traditional approaches with no standardised definitions in up to 19% of patients with tumours near the rectosigmoid junction [20].

### Lower boundary: anorectal junction and anal verge

**Fig. 4:**
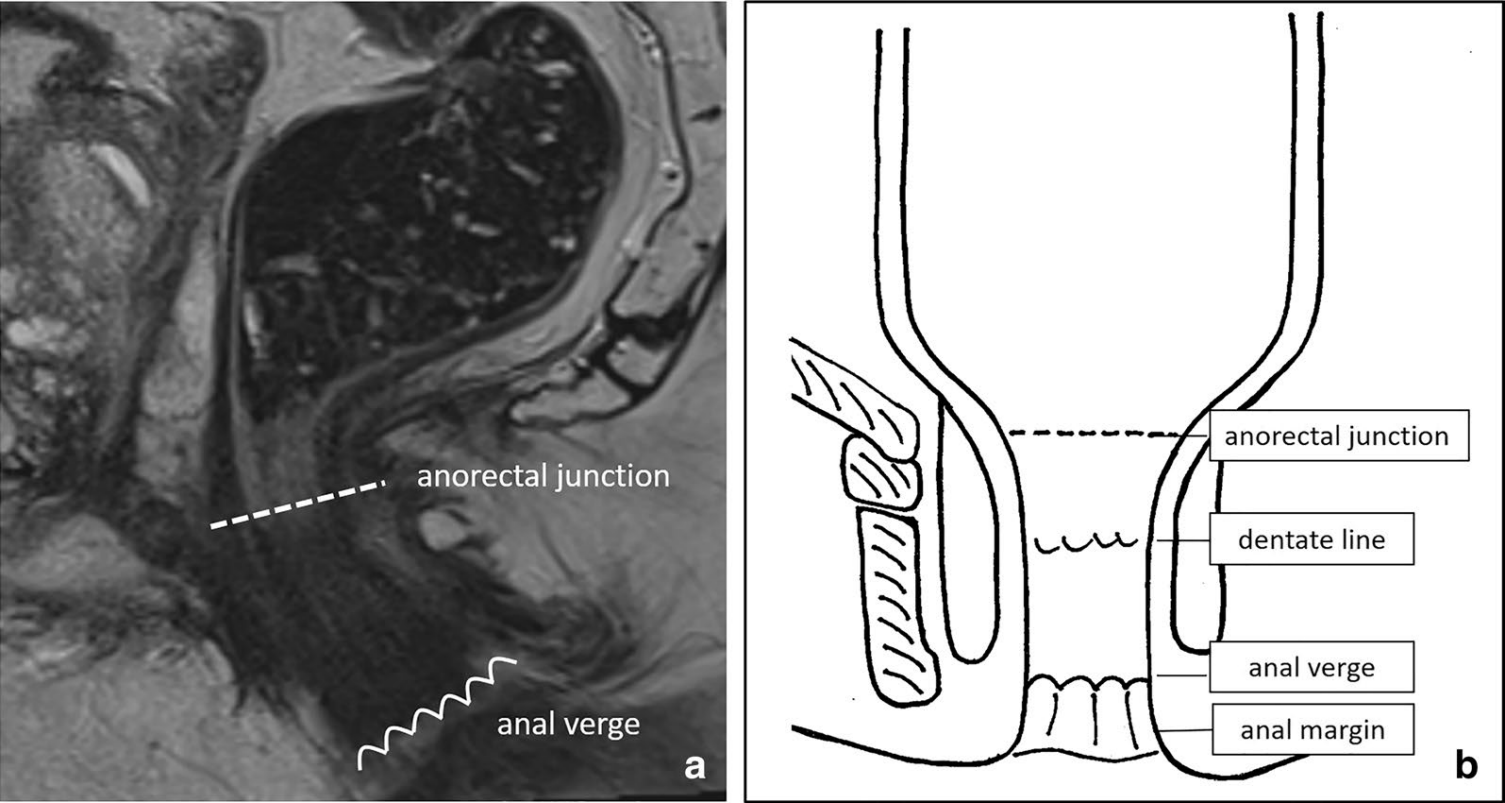
Sagittal T2-weighted MR image (**a**) demonstrating the normal anatomy of the anorectal junction (see also Fig. 2a) and the anal verge in a male individual without rectal cancer. Schematic coronal drawing (**b**) showing the anorectal junction and anal verge in relation to the dentate line (watershed junction between splanchnic and somatic innervation of the anorectum) and the anal margin, which are typically not very well appreciated on MRI

How to define the lower boundary of the rectum also remains a somewhat controversial topic. A commonly reported anatomical landmark is the dentate line, which marks the transition line between the columnar rectal mucosa and squamous anal mucosa. The dentate line is, however, not recognisable on imaging. Landmarks more commonly used in clinical practice are the anorectal junction and the anal verge (Fig. 4). The anorectal junction is commonly used by surgeons to separate the rectum from the anal canal. It is typically located 1–2 cm proximal to the dentate line and is palpable upon digital rectal examination at the level of the muscular anorectal ring, which includes the puborectal sling and the upper portions of the external anal sphincter [22, 23]. The anorectal junction can also be visualised with high reproducibility on MRI and is commonly used as a landmark from which to measure the height of the tumour, e.g. “tumour starts at … cm from the anorectal junction”. As a rule of thumb, on MRI the anorectal junction is situated at the level of an imaginary line between the lower margin of the sacral and pubic bone on sagittal MRI (Figs. 3A and 4) or on coronal plane as a line across the upper boundary of the puborectal sling. The anal verge marks the transition between the epithelium of the anal canal and the perianal skin. It is used by some radiologists instead of the anorectal junction as a landmark on MRI (Fig. 4) and is also typically used as a landmark during endoscopic examinations. The first ± 5 cm of perianal skin caudal to the anal verge is referred to as the anal margin. From a clinical point of view, defining the tumour's location (or height) is relevant because this information helps the surgeon determine whether or not there is sufficient margin between the lower border of the tumour and the anal canal to perform a low anterior resection and create an anastomosis. Ultimately this decision will be informed by a combination of digital rectal examination, endoscopy and MRI.

## The anal canal and pelvic floor

**Fig. 5:**
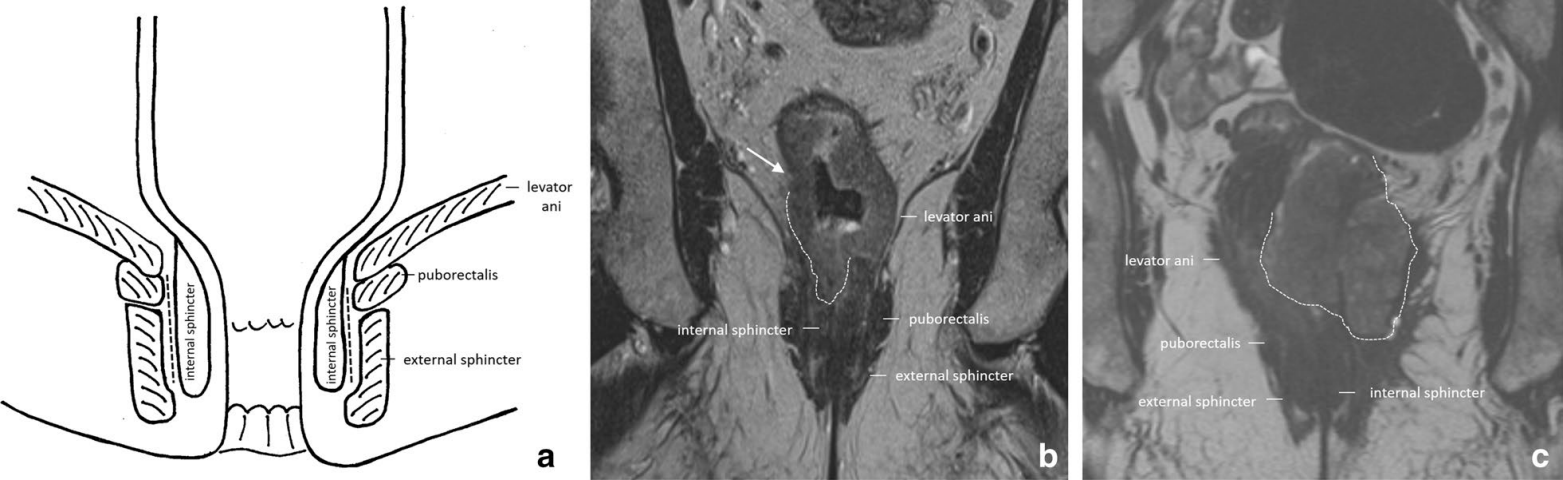
Schematic coronal drawing **a** showing the different layers of the anal canal and pelvic floor. The dotted line represents the intersphincteric plane. The coronal T2-weighted MRI in **b** is an example of a 62 year old male patient with a cT3a tumour (white arrow showing an irregular rectal wall with ± 1 mm perirectal extension) that extends into the anal canal and invades the right internal anal sphincter. The coronal image in **c** is an example of a 59-year-old female patient with a cT4b rectal tumour that invades the internal sphincter on both sides and extends into the external sphincter, levator ani, and puborectalis muscles on the left side

A good quality staging report of any low rectal tumour should include an accurate description of the relation of the tumour to the different layers of the anal canal and pelvic floor. The presence and extent of invasion into these respective structures define the surgical resection strategy and help the surgeon decide whether, for example, an intersphincteric resection could still be feasible or if the patient requires a routine or even extralevator abdominoperineal resection (APR; see also section on *surgical techniques* and *post-surgical anatomy* below). It is also important information for radiation oncologists to guide target volume delineation. To properly assess invasion into the anal canal and pelvic floor (Fig. 5), it is vital to include in the MRI protocol for low rectal tumours a high-resolution T2-weighted plane that is acquired parallel to the anal canal**.**

As shown in Fig. 5, the anal sphincter consists of three main layers. The inner layer, the ‘internal sphincter’, is a thickened continuation of the inner circular muscle of the rectum. The external sphincter is comprised of striated muscle and forms the outer part of the anal sphincter. It is continuous with the striated puborectalis and levator ani muscles at the upper end [24, 25]. Together with the iliococcygeus and pubococcygeus muscles, the puborectal and levator ani muscles form the “pelvic floor” [24, 25]. On MRI, the internal and external sphincter and pelvic floor muscles appear hypointense on T2W sequences, while the intersphincteric plane is typically hyperintense.

### Note: impact of anal sphincter and pelvic floor invasion on T-stage categorisation

With respect to T-stage categorisation of low rectal cancers, the American Joint Committee on Cancer (AJCC)/Union for International Cancer Control (UICC) Tumour Nodes Metastases (TNM) staging system does not clearly define how to take invasion of different layers of the anal sphincter and pelvic floor into account. In 2021, a multidisciplinary expert consensus panel of radiologists, surgeons, radiation oncologists, and pathologists discussed this issue. They proposed that the clinical T-stage (cT-stage) on MRI—like the pT-stage in pathology—should primarily be informed by the extent of tumour invasion at the level of the rectum. Involvement of the external sphincter, puborectalis, and/or levator ani muscles should be classified as cT4b disease as this entails skeletal muscle invasion. Invasion of the internal sphincter and intersphincteric plane by itself should not affect the cT-stage categorisation but their invasion should always be additionally described [26].

### Note: anal versus rectal cancer

It is important to note that the definition of rectal versus anal cancer primarily depends on the underlying tumour histology. Rectal cancers arise from large bowel mucosa and are typically adenocarcinomas, while anal cancers arise from squamous or transitional epithelium and are typically squamous cell carcinomas (SCC). Rectal adenocarcinomas may extend into the anal canal or may even be located for the majority within the anal canal. Conversely, anal squamous cell carcinomas may extend above the level of the anorectal junction and involve the rectum. SCCs originating primarily from the rectum and adenocarcinomas of the anal canal have also been reported but are rare, representing only a small minority of cases [27, 28]. Since the histological tumour type denotes important differences in tumour biology with subsequent differences in treatment responses, it is typically the main factor that guides clinical decision making, regardless of anatomical location and extension. Main differences in staging and treatment between rectal cancer and anal cancer are summarised in Table 2.

**Table 2:** Main differences in staging and treatment stratification between anal and rectal cancer

	Anal cancer	Rectal cancer
Typical histology	Squamous cell carcinoma	Adenocarcinoma
Treatment	– Low risk (T1-stage): local excision or local radiotherapy	– Low risk: surgery only (total mesorectal excision)
	– High risk (≥ T2-stage): definitive chemoradiotherapy	– Intermediate and high-risk: neoadjuvant (chemo)radiotherapy
T-stage definitions^a^	Primarily based on size (largest dimension):	Primarily based on depth of invasion:
	T1—tumour ≤ 2 cm	T1—tumour invades submucosa
	T2—tumour > 2 cm but ≤ 5 cm	T2—tumour invades muscularis propria
	T3—tumour > 5 cm	T3—tumour invades perirectal fat
	T4—tumour of any size that invades adjacent organs	T4—tumour invades peritoneum (T4a) or adjacent organs/structures (T4b)
N-stage definitions^a^	Primarily based on location of regional N+ nodes:	Primarily based on number of regional N + nodes:
	N0 – no N+ nodes	N0 – no N+ nodes
	N1a – N+ nodes in inguinal, mesorectal and/or internal iliac (including obturator) regions	N1 – 1–3 N+ nodes
	N1b – N+ nodes in external iliac region	N2 – ≥ 4 N+ nodes
	N1c – N+ nodes in N1a and N1b regions	

CRT = Chemoradiotherapy; TME = total mesorectal excision

^a^Definitions based on 8th edition of AJCC/UICC tumour node metastases (TNM) staging manual

## The mesorectal compartment, mesorectal fascia and peritoneum

### Mesorectum and mesorectal fascia

**Fig. 6:**
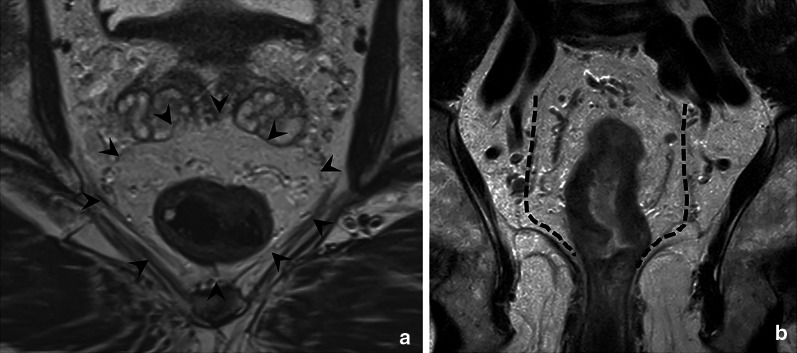
T2-weighted axial MR image **a** of a male individual demonstrating the mesorectal fascia as a thin hypointense line (arrowheads) surrounding the mesorectal compartment. Coronal image **b** demonstrating the distal tapering of the mesorectum

The rectum and surrounding mesorectal fat (the mesorectum) are enveloped by the mesorectal fascia (MRF) and are fixed to the sacrum by the presacral fascia of Waldeyer. The MRF is a thin fibrous structure that comprises the anticipated resection plane when performing a total mesorectal excision (TME). The term MRF is sometimes used interchangeably with the term circumferential resection margin (CRM), which is incorrect. The MRF is an anatomical structure, whereas the CRM is a more technical term indicating the margin a surgeon creates when performing his resection (and the margin pathologists report when describing the smallest distance between the tumour and the outer plane of the resected specimen). Therefore, radiologists should avoid using CRM but rather describe the tumour in relation to the MRF [29]. The MRF should be considered as involved when the tumour invades the MRF directly or the margin between the tumour and MRI is ≤ 1 mm (Fig. 1) [2]. In a recent expert consensus guide it was proposed that these criteria apply to the primary tumour, but also to EMVI or any irregular nodes or tumour deposits that invade or are within 1 mm from the MRF [26]. On T2-weighted MRI, the MRF is easily recognised as a thin hypointense line surrounding the mesorectum (Fig. 6A). The mesorectal fat is thinner on the anterior side than on the lateral and posterior sides. Therefore, a close relation exists between the anterior rectal wall and the prostate and seminal vesicles in men and the vagina and cervix in women. The mesorectal compartment tapers towards the distal end (Fig. 6B). Consequently, tumours located in the distal rectum are at higher risk for MRF involvement. In case of suspected MRF involvement, patients are typically stratified for neoadjuvant treatment aiming to induce tumour downsizing, increase the chance of a tumour free resection margin and reduce the chance of a local recurrence.

### Peritoneum and peritoneal reflection

**Fig. 7:**
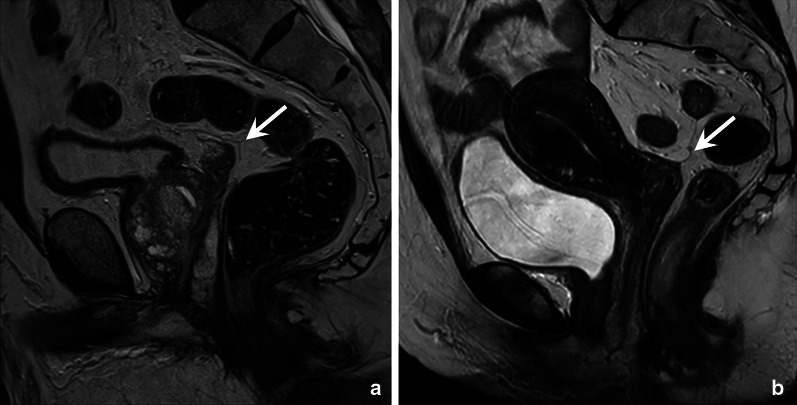
Sagittal T2-weighted MR images demonstrating the anterior peritoneal reflection in a male individual (without rectal cancer), at the top of the seminal vesicles (**a**) and in a female (without rectal cancer) where it is located at the level of Douglas’ pouch (**b**). The peritoneal reflection can be recognised as a thin V-shaped “fold” (arrows)

The anterior peritoneal reflection is a thin layer of visceral peritoneum that separates the rectum’s extra- and intraperitoneal parts. On MRI, it typically has a V-shaped appearance (or gull-wing appearance, therefore sometimes referred to as the “seagull sign”). In the sagittal plane, it extends from the top of the seminal vesicles in men and from the level of the cul-de-sac (Douglas’ pouch) in women, as shown in Fig. 7 [30].

**Fig. 8:**
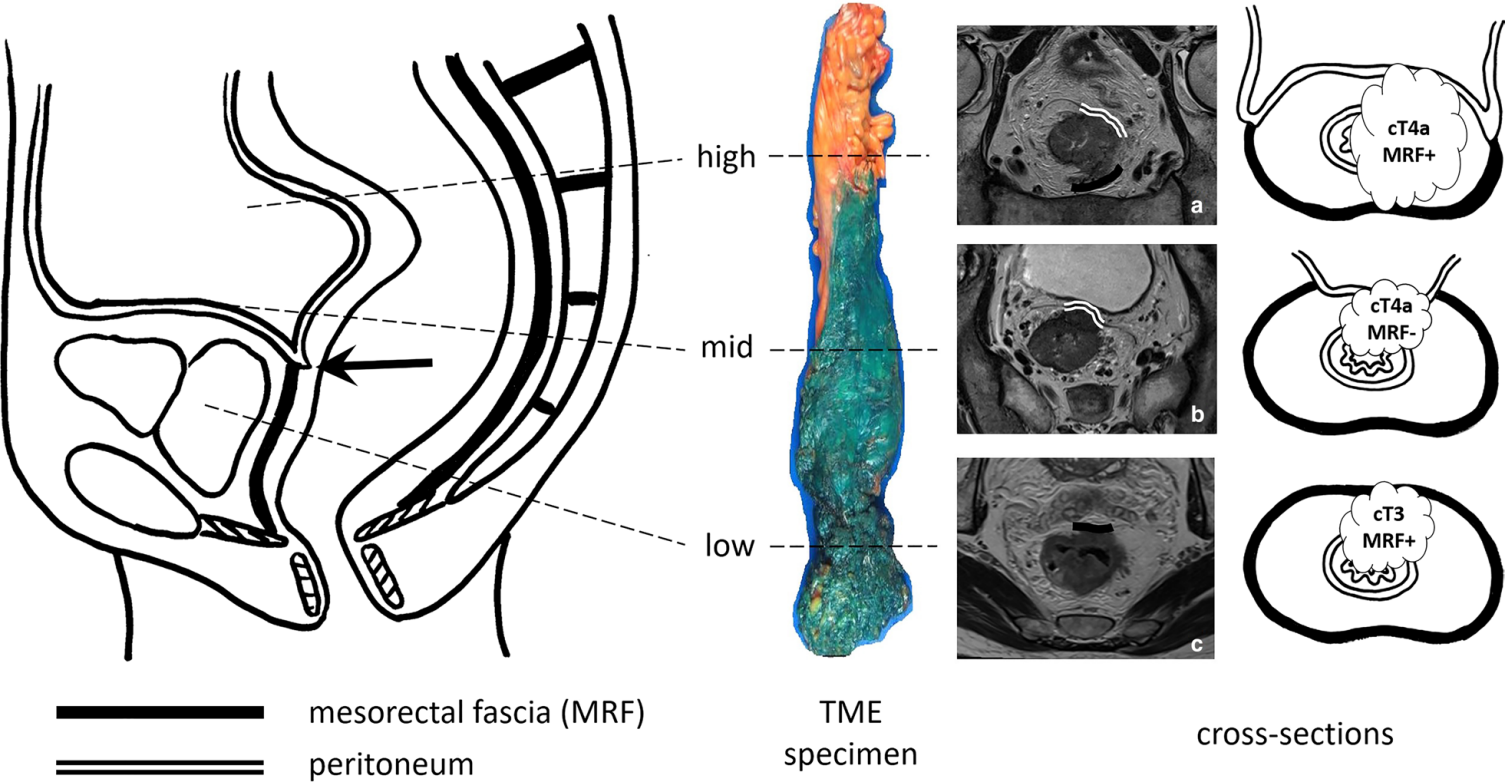
Schematic drawing and total mesorectal excision (TME) resection specimen showing the mesorectal fascia (inked in green in TME specimen) extending up until the level of the peritoneal reflection (black arrow) anteriorly. From this level upwards, the mesorectum is covered by peritoneum anteriorly in the mid-rectum, and anterolaterally in the high rectum. Invasion of the peritoneum including the anterior peritoneal reflection entails cT4a disease (**a**, **b**). Invasion of the MRF is classified as cT3 MRF + disease. Note that invasion of the MRF may co-occur with invasion of the peritoneum, in which case the tumour stage is cT4a MRF + (**a**)

It is important to realise that the MRF envelopes the entire circumference of the mesorectal compartment only below the level of the anterior peritoneal reflection. Above this level, the MRF ascends dorsolaterally to cover the mesorectum only on the lateral and dorsal part. Above the peritoneal reflection, the anterior mesorectum is covered by peritoneum, as illustrated in Fig. 8. The MRF and peritoneum are thus two separate anatomical structures, and separate recognition of their respective invasion is important for cT-staging. While invasion into the MRF constitutes cT3 disease (cT3 MRF+), invasion of the peritoneal covering at any location, including the anterior peritoneal reflection, constitutes cT4a disease. When MRF invasion and peritoneal invasion co-occur, this entails cT4a MRF+ disease, as shown in Fig. 8 [2].

## Extramesorectal organs and ‘structures’

When staging locally advanced tumours, it is essential to clearly describe any tumour invasion into organs or structures in the pelvis situated outside the mesorectal compartment. This information is important for surgical and radiotherapy planning, but also impacts the cT-stage classification. Note that the AJCC/UICC TNM staging system does not include a clear description of what is covered by the umbrella term “structures” when classifying T4b disease as “any tumour with invasion of another organ or structure”. The lack of a clear definition for cT4b disease was identified as an important staging controversy by a recently published survey on the radiological application of the TNM staging system for rectal cancer [15, 26]. Based on the outcomes of this survey, a multidisciplinary panel of experts agreed that from a treatment point of view, cT4b disease should include any tumour with direct invasion of either another organ or any anatomical compartment or structure outside the mesorectum on MRI that would require adaptation of the standard surgical resection plane, including (see also T-stage definitions in Table 1):

- Pelvic organs (uterus, ovaries, vagina, prostate, seminal vesicles, bladder)
- Bone
- striated/skeletal muscle (incl. external anal sphincter, puborectalis, and levator ani, obturator, piriformis, and ischiococcygeus)
- ureters and urethra
- sciatic or sacral nerves
- sacrospinous/sacrotuberous ligaments
- any vessel outside the mesorectal compartment
- any loop of small or large bowel in the pelvis (separate from the primary site from which the tumour originates)
- any fat in an anatomical compartment outside the mesorectal compartment (i.e. obturator, para-iliac, or ischiorectal space)

**Fig. 9:**
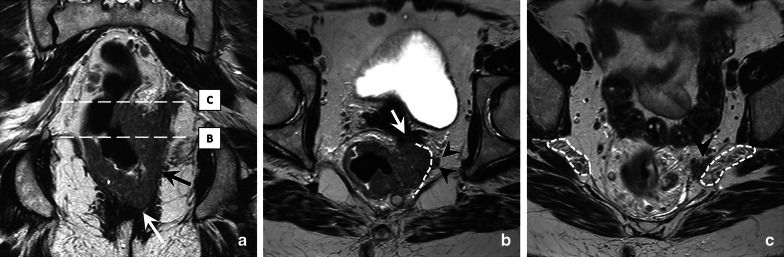
Coronal T2-weighted image (**a**) and axial T2-weighted cross sections at two different levels (**b**, **c**) of a 48 year old female patient with a cT4b rectal tumour based on invasion of the left external anal sphincter (white arrow in **a**; = skeletal muscle invasion), invasion of the pelvic floor (black arrow in **a**; = skeletal muscle invasion), invasion of the obturator compartment (arrowheads in **b**; = invasion of compartment outside the mesorectum), and invasion of the vagina (white arrow in **b**; = organ invasion). There is also a close relation to the sacral nerve plexus (white dotted lines) on the left side (arrowhead in **c**)

Different examples of cT4b invasion are provided in Fig. 9. Note that invasion of the peritoneum alone—even though situated outside the mesorectal compartment—is not considered cT4b disease but is classified separately as cT4a as detailed above.

## Blood supply, lymphatic drainage and lymph node stations

### Vascular supply

**Fig. 10:**
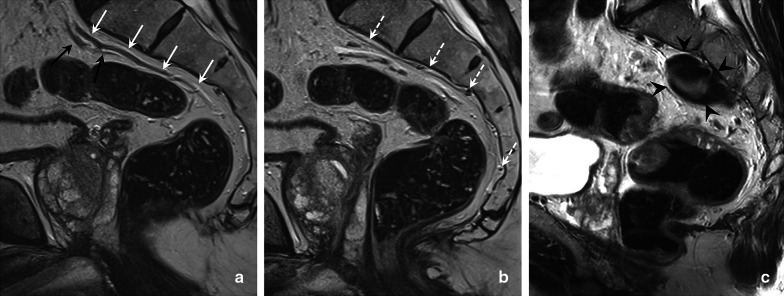
Sagittal images of a male individual without rectal cancer (**a**, **b**) demonstrating the superior rectal artery (black arrows in **a**) and superior rectal vein (white arrows in **a**) that form the main blood supply of the rectum. The dashed arrows in **b** show the small venous structures that form the presacral venous plexus situated outside the mesorectal compartment behind Waldeyer’s fascia. The sagittal image in **c** shows a different case where the presacral venous plexus is severely dilated (black arrowheads)

The arterial supply of the rectum consists of the superior, middle and inferior rectal arteries. The superior rectal artery is the terminal branch of the inferior mesenteric artery and constitutes the main feeding artery of the rectum. On axial T2-weighted MR images, the superior rectal artery and its branches can be detected as low-intensity, tubular structures in the presacral region (Fig. 10A), with the superior rectal vein running parallel to it (typically on the left dorsal side). The distal part of the rectum receives additional blood supply from the middle rectal artery, an inconsistent branch from the internal iliac artery. The inferior rectal artery originates from the pudendal artery, a branch of the internal iliac artery. It is situated below the pelvic floor muscles and contributes very little to the blood supply of the rectum; it mainly supplies the distal part of the anal canal. Anastomoses between the lateral and median sacral veins, which accompany the corresponding arteries that arise from the dorsal side of the aorta just above the bifurcation, together form the so-called presacral venous plexus behind Waldeyer’s fascia (Fig. 10B, C). The presacral venous plexus can bleed profusely if accidentally injured during rectal surgery.

Note that the upper two-thirds of the rectum are drained by the superior rectal vein, which empties into the portal system via the inferior mesenteric vein. The venous drainage of the lower third of the rectum runs via the middle and inferior rectal veins, which drain into the systemic venous circulation via the internal iliac veins [31]. This explains why lower rectal tumours have a relatively higher incidence of pulmonary metastases (without hepatic metastases) than higher tumours [31].

### Small mesorectal vasculature and extramural vascular invasion (EMVI)

**Fig. 11:**
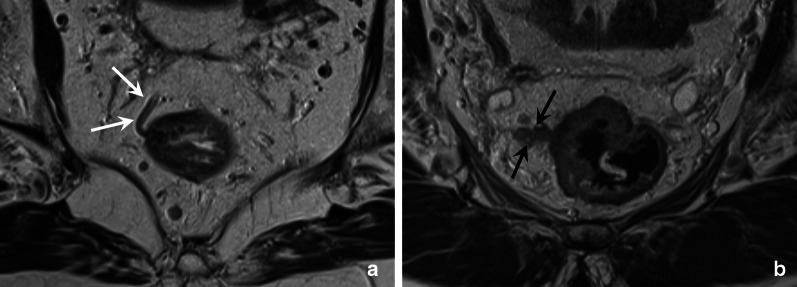
Axial T2-weighted images of two rectal cancer patients; a 63-year-old male patient with a cT1-2 rectal tumour (**a**; tumour not shown) an another 83-year-old female patient with a cT3cd tumour (**b**). The image in A is a cross section just above the level of the tumour in the rectal wall and shows a small vessel radiating outwards from the muscularis propria into the perirectal fat (white arrows). The vessel has a normal contour and low T2 signal; there are no signs of EMVI. The image in **b** shows a semicircular tumour that involves the rectal wall from 7 till 1 o’clock. The intermediate signal of the tumour extends into the adjacent vessels. The vessels is expanded, and the vessel contour is disrupted. These are all signs indicative of EMVI

In addition to the larger arteries and veins described in the previous section, there are also numerous unnamed smaller vessels that radiate outwards from the edge of the muscularis propria into the perirectal fat. These smaller vessels can be visualised as small serpiginous branches, as illustrated in Fig. 11A. Extramural vascular invasion (EMVI) can occur in tumours that grow beyond the muscularis propria (≥ T3) and is defined as the extension of tumour within these small perirectal blood vessels [32]. It is a known adverse prognostic risk factor associated with recurrent disease, metastases and impaired overall survival [33]. On MRI, EMVI can be visualised as direct tumour signal extending into a blood vessel, with or without expansion of the vessel or infiltration of the vessels borders (Fig. 11B).

### Lymphatic drainage and lymph node stations

The main lymphatic drainage of the rectum follows the superior rectal artery and vein towards the inferior mesenteric vein. Most of the lymph nodes in the mesorectum are situated along these vessels in the posterior and lateral parts of the mesorectum [34]. Criteria used in current guidelines to characterise mesorectal lymph nodes as malignant are based on a combination of size and morphology. Nodes are considered as suspicious for N+ when ≥ 9 mm, 5–8 mm with two morphologically suspicious features, or < 5 mm with three suspicious features. Morphologic criteria suspicious for malignancy are an indistinct border, round (rather than oval) shape, and heterogeneous signal. Mucinous lymph nodes (in mucinous tumours) are always considered as cN+ [2].

**Fig. 12:**
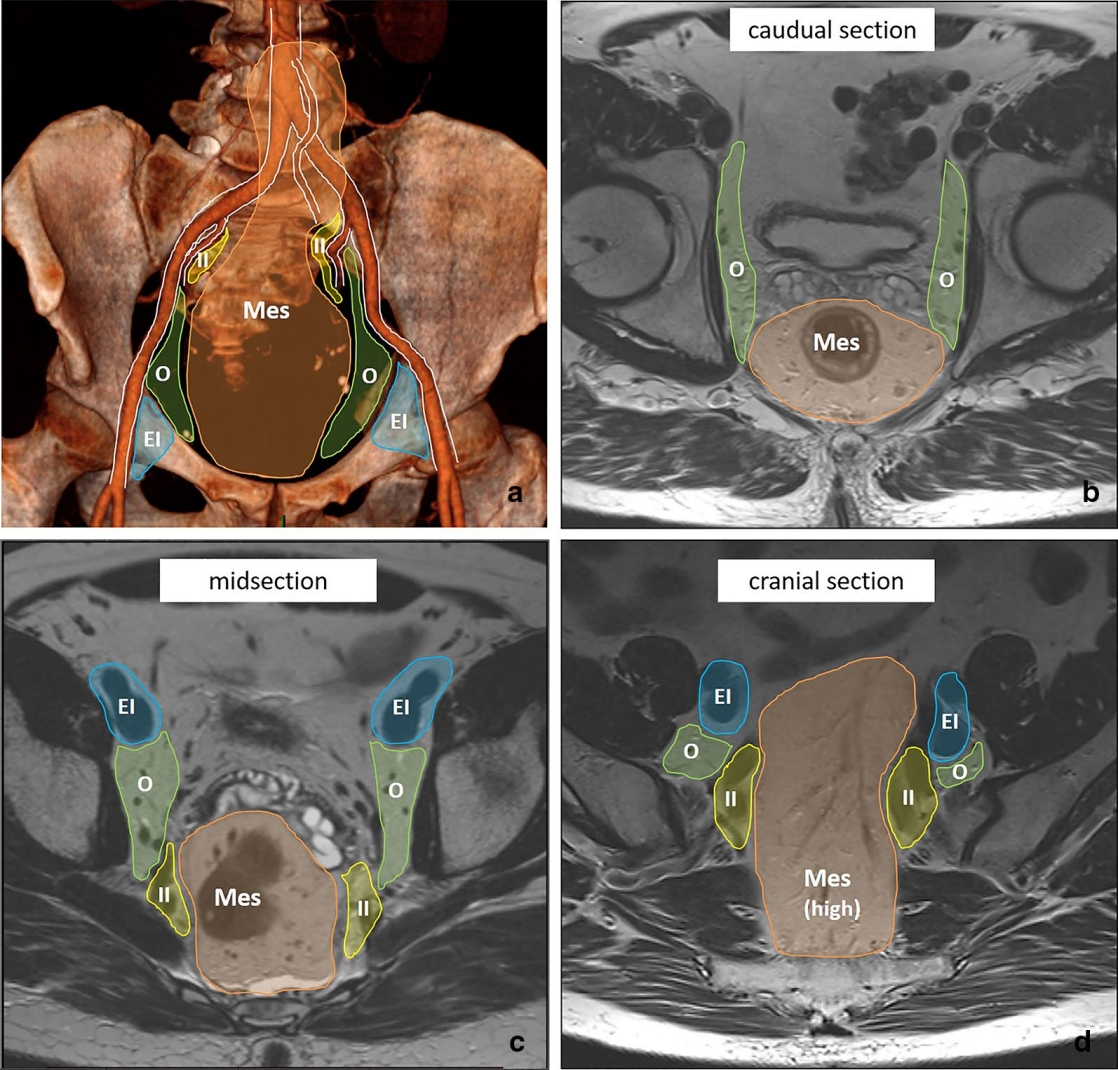
Overview of pelvic lymph node stations on MRI (**a**) with corresponding axial cross sections at the caudal (**b**), mid (**c**) and cranial (**d**) level. The mesorectal lymph nodes (Mes) in orange include all nodes in the mesorectal compartment, including the high mesorectal nodes that follow the superior rectal artery and vein towards the inferior mesenteric vein. The obturator nodes in green, are located dorsal from the external iliac vein and lateral from the lateral border of the main trunk of the internal iliac vessels, that separate the obturator (O) from the internal iliac (II) compartment in yellow. The external iliac nodes (EI) in blue are located alongside the external iliac vessels

Tumours below the anterior peritoneal reflection (in the distal and middle parts of the rectum) follow an additional lymphatic drainage route alongside the middle rectal artery and vein towards the so-called lateral nodal stations situated outside the mesorectum. These lateral nodes include the internal iliac, obturator and external iliac nodes [35–37]. Note that in some publications (mainly from Japanese studies), the common iliac nodes are also referred to as lateral nodes [38, 39]. Pathologic lymph nodes in the obturator and internal iliac areas are—despite their extra-mesorectal location—still considered “regional” disease and are mainly associated with an increased risk for lateral local recurrence [35, 37, 40]. Though included as regional nodes in the N-stage category of the TNM staging system, pathologic obturator and internal iliac nodes need to be reported separately as they will not be removed with standard TME surgery and require targeted radiotherapy and/or lateral nodal dissection to avoid lateral nodal recurrences. Radiologists thus need to alert the radiation oncologist and surgeon of any N+ nodes in these regions to guide target delineation and surgical planning. Data from the Lateral Nodal Study Consortium indicate a short axis diameter of ≥ 7 mm as a criterion to diagnose N+ nodes in the obturator and internal iliac regions; unlike in mesorectal lymph nodes, morphologic criteria are not of added benefit for lateral nodal staging [36, 37]. Nodal metastases along the external and common iliac vessels are much less common and are mainly associated with an increased risk for distant metastases [40]. Therefore, these nodes are considered non-regional disease and included in the M-stage classification. Pathologic inguinal nodes also constitute M+ disease, although—like in anal cancer—they may still be considered regional nodal metastases in tumours extending into (or situated primarily in) the distal anal canal, considering the regional lymphatic drainage route from the anal canal towards the superficial inguinal nodes [15]. Table 3 provides an overview of the different lymph node stations and variations in terminology used to describe them, which can sometimes be a source of confusion. Clear guidelines describing how to discern the different lymph node compartments on imaging have also been lacking, which has contributed to substantial variation in the radiological reporting of these nodes [40]. Figure 12 details how the various mesorectal and lateral lymph node stations can be discerned on MRI using surgical definitions derived from a publication by Ogura et al. from 2019 [37]. These definitions can serve as a roadmap for radiologists to help improve consistency in nodal reporting.

**Table 3:**
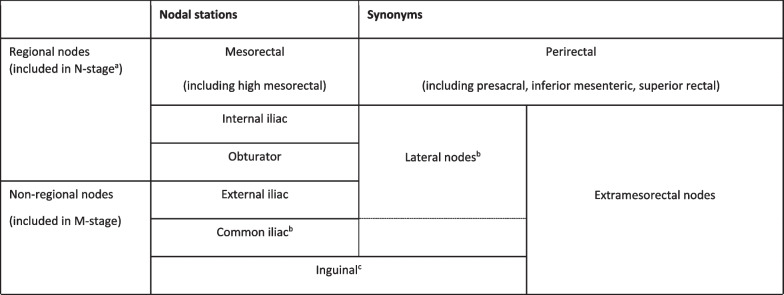
Terminology to describe regional and non-regional pelvic lymph nodes for rectal cancer staging

^a^N0 = no regional N + nodes, N1 = 1–3 regional N + nodes (N1a = 1; N1b = 2–3), N2 =  ≥ 4 regional N + nodes (N2a = 4–6; N2b =  ≥ 7)

^b^The “lateral nodes” typically include the internal iliac, obturator and external iliac nodes. Note that in some (mainly Japanese) publications, the common iliac nodes are also referred to as “lateral nodes”

^c^In distal tumours extending into (or situated primarily in) the distal anal canal, inguinal nodes may still be considered as regional (N-stage) nodes, considering the regional lymphatic drainage route from the anal canal

## Anatomical considerations after neoadjuvant treatment

**Fig. 13:**
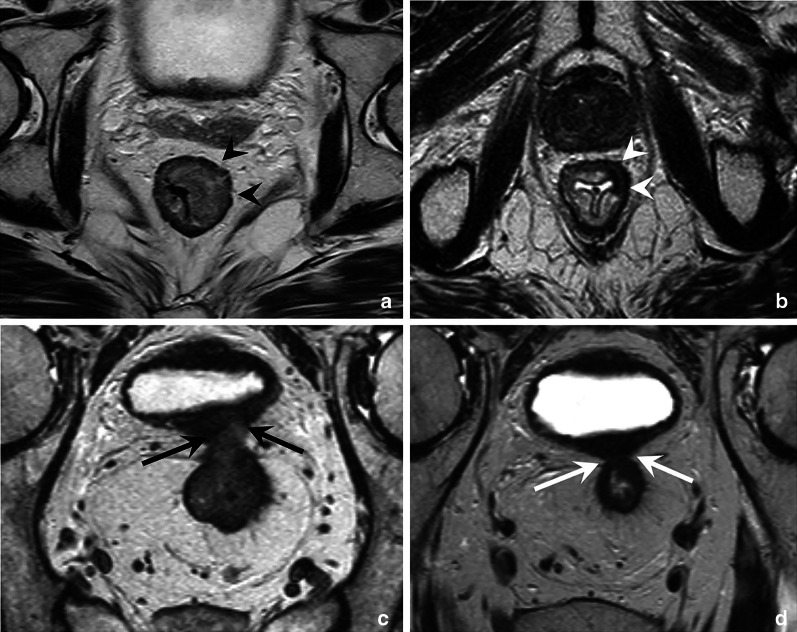
Pre-treatment and post-chemoradiotherapy T2-weighted MR images of 74-year-old male patient with a distal tumour primarily staged as cT1-2 (upper row; **a** = pre-treatment, **b** = post-treatment, arrowheads indicating intact bowel wall around the tumour), and another 62 year old male patient with a more advanced cT4b tumour invading the dorsal bladder wall (bottom row, **c** = pre-treatment, **d** = post-treatment, arrows indicating invasion of the bladder). In both cases the tumour has decreased in size and become largely hypointense after CRT, indicating a fibrotic transformation (white arrowheads in **b**, white arrows in **d**). If and to what extent viable residual tumour is present within the fibrosis is difficult to discern. The upper patient proved to be a complete responder (ycT0, followed by watch-and-wait with no signs of tumour regrowth for > 2 years). The bottom patient underwent resection showing a ypT3 tumour remnant at histopathology. The fibrosis invading and retracting the bladder wall (white arrows in **d**) did not contain any vital residual tumour cells

Commonly used neoadjuvant treatment regimens include a short course of radiotherapy (5 × 5 Gy) prior to TME for intermediate-risk tumours (typically cT3cd and/or cN+) and a long course of combined chemoradiotherapy for locally advanced tumours (typically cT3 MRF+, cT4 and/or cN+). The latter is intended to induce tumour downsizing and downstaging to enhance the chance of radical surgical resection [4, 14]. In response to these treatments, rectal tumours typically decrease in size while undergoing a fibrotic transformation. When tumours become fibrotic, their T2-weighted signal drops from intermediate (lower than fat, higher than muscle) to markedly hypointense, as illustrated in Fig. 13. When the tumour bed has become predominantly fibrotic, it is difficult to discern on T2-weighted MRI whether we are dealing with only fibrosis or fibrosis still containing nests of viable residual tumour. This greatly limits the performance of standard MRI in the restaging setting, resulting in a suboptimal performance for ycT-staging, but also for and assessment of yEMVI and yMRF involvement [41–43]. Diffusion-weighted imaging (DWI) highlights hypercellular tissues and can enhance the performance of MRI to detect areas of vital (hypercellular) residual tumour within the fibrotically changed tumour bed. DWI is therefore now recommended to be included in the standard MRI protocol for restaging after neoadjuvant treatment, in specific for the differentiation between complete responders (who may be candidates for organ preservation) and patients with residual tumour [2]. Evidence on the benefit of DWI for further restaging (e.g. yEMVI, yMRF, yN) is limited [44]. In addition, specific imaging patterns and criteria have been described in the restaging setting, such as the MRI tumour regression grade (mrTRG) to grade the degree of fibrosis versus residual tumour, or patterns to help assess the risk of persistent MRF invasion in case of fibrosis after CRT. An in-depth discussion of these patterns, the pearls and pitfalls of DWI, and the role of MRI for organ preservation is, however, outside the scope of this anatomy-focused review. We therefore kindly refer the interested reader to previous publications on these topics [41, 44–47].

## Surgical techniques and post-surgical anatomy

**Fig. 14:**
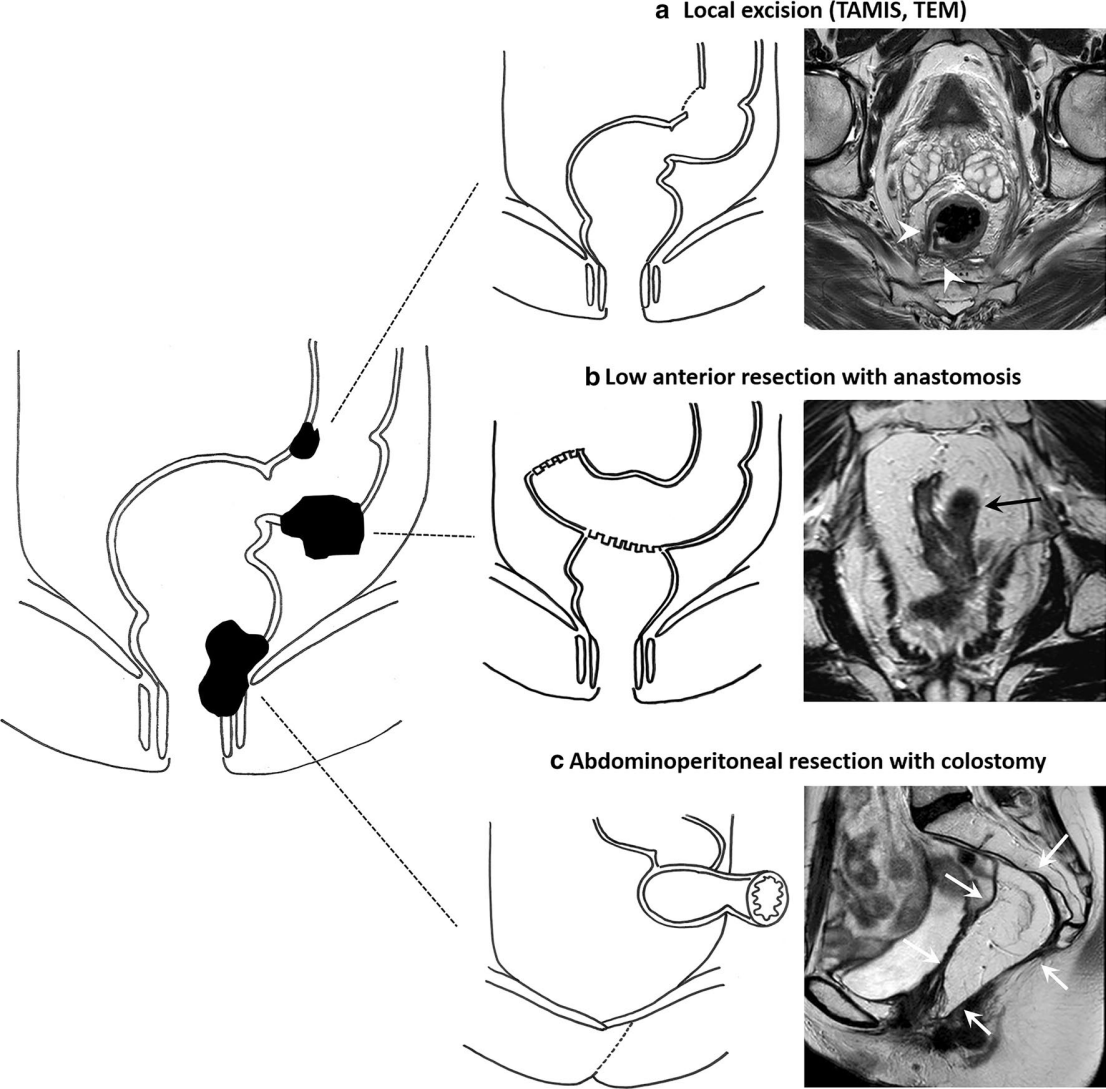
Schematic illustration showing different surgical techniques used to resect rectal cancer, including their post-operative appearance on MRI. With TAMIS and TEM (**a**), a full-thickness resection of the tumour and the rectal wall results in a focal wall defect and surrounding fibrotic changes on post-operative MRI (white arrowheads). After a low anterior resection (**b**), patients typically receive a ‘side-to-end’ anastomosis where the sidewall of the proximal colon loop is anastomosed to the end of the rectum stump, creating a small blind-ending loop of the colon that can also be recognised on post-operative MRI (black arrow). After an abdominoperineal resection, the rectum and anal canal are no longer in situ, and the patient receives a permanent colostomy. In this case, the post-operative defect in the pelvis and pelvic floor was reconstructed with a myocutaneous rectus muscle flap (white arrows)

MRI is not routinely performed during the follow-up of patients after curative resection. However, it is valuable (as a second-line modality) to help detect and evaluate the extent of disease in patients with suspected pelvic recurrence. In these cases, a proper understanding of post-surgical anatomy is crucial. A schematic overview of some of the most commonly used surgical techniques, including corresponding post-surgical MR images, is provided in Fig. 14.

### Local excision

Local excision is a general term used to describe minimally invasive endoscopic techniques used to resect non-cancerous polyps and early-T-stage cancers (T1 and some good prognostic T2 tumours). Endoscopic mucosal resection (EMR) and endoscopic submucosal resection (ESD) are superficial excision techniques, while transanal minimally invasive surgery (TAMIS) or transanal endoscopic microsurgery (TEM; a similar but older technique) allow full-thickness resection of the rectal wall up to the mesorectal fat. After EMR or ESD, MRI can show a subtle focal fibrotic scar at the excision site, although no abnormalities are observed in many cases. After TEM/TAMIS, MRI typically shows a defect in the rectal wall surrounded by fibrosis (Fig. 14A). Early post-operative changes may also include inflammatory changes and oedema, which can give the former tumour bed and scar a very irregular appearance that should not be mistaken for recurrence [48]. After a more extended follow-up period, these inflammatory changes gradually disappear.

### Total mesorectal excision (TME)

TME remains the standard surgical procedure for rectal cancer. With TME, the entire mesorectal compartment is removed alongside the MRF. In upper rectal or rectosigmoid junction tumours, a partial mesorectal excision (PME) can be performed where part of the distal-middle rectum and mesorectum are left in situ. TME is an umbrella term that covers different surgical resection techniques, including a low anterior resection (LAR), and an abdominoperineal resection (APR). LAR is typically performed in middle-upper tumours. The anal canal is left in situ, and there is sufficient margin to create an anastomosis (typically a ‘side-to-end’ anastomosis) between the remaining distal rectum and sigmoid colon (Fig. 14B). APR is indicated for low rectal tumours that approximate or involve the anal canal. With an APR, the rectum and anal canal are resected en bloc, and the patient receives a permanent colostomy (Fig. 14C). Variations to the standard APR include the intersphincteric approach, where the external sphincter is spared, and the extralevator APR, a more extensive procedure for tumours invading the pelvic floor, including resection of the levator ani muscles.

**Fig. 15:**
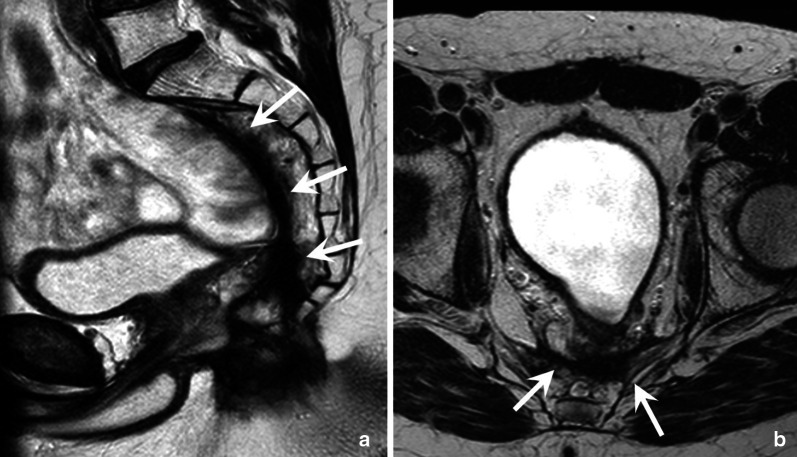
Sagittal (**a**) and axial (**b**) T2-weighted images of a 41-year-old male patient who underwent an abdominoperineal resection for a ypT3N2 low rectal tumour. Note the extensive post-operative fibrotic changes (white arrows). These are part of the normal post-operative spectrum and should not be mistaken for residual or recurrent tumour

After APR, the pelvic floor and perineum can be closed primarily, with the use of a mesh, or with plastic reconstructive techniques such as the vertical or oblique rectus abdominis myocutaneous flaps (VRAM/ORAM, see example in Fig. 14C), or gluteal flaps. Additionally, the greater omentum (omentoplasty) can be used to fill the pelvis. Early post-operative T2-weighted images of the muscular portion of a musculocutaneous flap show (low) muscle signal intensity. Over time, denervation results in muscular atrophy, and eventually, the muscular part of the flap is replaced by fat with a corresponding increase in signal (see Fig. 14C) [49]. An omentoplasty also shows a high signal on T2-weighted MRI as it primarily contains fat. Some small lymph nodes may be present within the omentoplasty that will typically be easy to recognise as benign (smooth, oval, homogeneous with fatty hilum) [46]. Other common findings after TME, especially in patients who have experienced post-operative anastomotic leakage, include the formation of extensive post-operative fibrosis and sometimes chronic presacral sinus formation [50]. An example of a case with extensive post-operative fibrosis is given in Fig. 15. These fibrotic changes should not be mistaken for residual or recurrent tumour.

## Conclusions

With this pictorial review, we aimed to provide an image-based overview of key anatomical concepts essential for treatment planning, response evaluation and post-operative assessment on MRI. A good understanding of the MRI anatomy of the rectum and its surroundings is pivotal to ensure high-quality diagnostic evaluation and reporting for primary staging and treatment planning of patients with rectal cancer. Knowing the spectrum of normal changes in anatomy and morphology of the rectal wall following (chemo)radiotherapy and key surgical concepts are vital to understanding how to interpret MRI following neoadjuvant or curative surgical treatment.

## Data Availability

Data sharing is not applicable to this article as no datasets were generated or analysed during the current study.

## Abbreviations

AJCC: American Joint Committee on Cancer
APR: Abdominoperineal resection
CRM: Circumferential resection margin
CRT: Chemoradiotherapy/chemoradiation
DWI: Diffusion-weighted imaging
EMR: Endoscopic mucosal resection
ESD: Endoscopic submucosal resection
ESGAR: European Society of Gastrointestinal and Abdominal Radiology
LAR: Low anterior resection
MRF: Mesorectal fascia
MRI: Magnetic resonance imaging
PME: Partial mesorectal excision
SCC: Squamous cell carcinoma
STO: Sigmoid take-off
T2W: T2-weighted
TAMIS: Transanal minimally invasive surgery
TEM: Transanal endoscopic microsurgery
TME: Total mesorectal excision
T-stage: Tumour stage
UICC: Union for International Cancer Control
